# Genetics of Sudden Cardiac Death

**DOI:** 10.3390/diseases14010007

**Published:** 2025-12-27

**Authors:** Martina Lovrić Benčić, Rea Levicki

**Affiliations:** 1 Department of Cardiovascular Diseases, University Hospital Centre Zagreb, School of Medicine, University of Zagreb, 10000 Zagreb, Croatia; martina.lovric-bencic@kbc-zagreb.hr; 2 Department of Cardiology, Požega General Hospital, 34000 Požega, Croatia

**Keywords:** sudden cardiac death, genetics, sudden arrhythmic death, genetic cardiomyopathy

## Abstract

Introduction: Cardiomyopathies (DCM, HCM, and ACM) and primary arrhythmogenic disorders (BrS, LQTS, and CPVT) represent the most common causes of sudden cardiac death (SCD) in young individuals. Systematic genome-wide single-nucleotide polymorphism (SNP) analyses and genome-wide association studies (GWASs) have enabled the identification of numerous genetic variants associated with cardiovascular diseases. Body: Genetic testing for cardiomyopathies and inherited channelopathies primarily involves panel testing of genes with definitive and strong evidence of disease association; genes supported by moderate evidence may also be considered. Cardiomyocytes express a variety of proteins implicated in the pathogenesis of genetic cardiomyopathies, including sarcomeric, cytoskeletal, desmosomal, and nuclear envelope proteins. Inherited cardiac channelopathies result from mutations in genes encoding cellular components that influence calcium ion availability or affect membrane ion channels, including sodium, potassium, and calcium channels. Common variants associated with SCD are found in genes encoding cardiac ion channels (e.g., *SCN5A*, *KCNQ1*, and *KCNH2*), calmodulin (*CALM2*), sarcomeric proteins (*MYH7*, *MYBPC3*, *TTN*, and *TNNI3*), and desmosomal proteins (*RyR2* and *DES*). Conclusions: This review demonstrates that specific genetic variants are significantly associated with an increased risk of SCD. The evidence underscores the importance of genetic screening and early intervention in individuals with a family history of SCD or other risk factors for inherited cardiac disorders predisposing to SCD. Future research should focus on gene-specific management strategies for familial cardiomyopathies and inherited channelopathies, with the goal of improving targeted genetic therapies and reducing the burden of sudden cardiac death.

## 1. Introduction

Sudden cardiac death (SCD) is defined as a sudden, natural death of cardiac origin that occurs within one hour of symptom onset in witnessed cases, or within 24 h of last being seen alive when unwitnessed. Autopsy findings often confirm SCD as an unexpected natural death of cardiac or unknown cause [1]. Cardiomyopathies (dilated cardiomyopathy (DCM), hypertrophic cardiomyopathy (HCM), and arrhythmogenic cardiomyopathy (ACM)) represent the most common causes of sudden cardiac death in adults under 40 years of age. However, in the pediatric and adolescent populations, primary arrhythmogenic disorders such as Brugada syndrome (BrS), long QT syndrome (LQTS), and catecholaminergic polymorphic ventricular tachycardia (CPVT) are more prevalent causes of SCD [2]. The incidence of sudden cardiac death increases with age. In individuals in their forties, coronary artery disease becomes the leading cause, while structural heart disease predominates in older populations [3,4]. Across all age groups, the incidence of SCD is consistently higher in men than in women [5].

The Atherosclerosis Risk in Communities (ARIC) study investigated racial differences in the cumulative risk of SCD, which significantly influence susceptibility, with individuals of African ancestry exhibiting a higher risk than those of European ancestry [6]. In a prospective study of children and young adults (Australia and New Zealand), 490 cases of SCD were identified, with an annual incidence which was 1.3 cases per 100,000 persons (aged 1–35 years; 72% male). The most frequent explained cause of SCD was coronary artery disease (24% of all SCD under 35 yrs), followed by inherited cardiomyopathies (16%) [7]. The most common cause of death in individuals younger than 31 years was unexplained SCD accounting for 40% of cases. Clinically relevant cardiac gene mutation was identified in 27% of unexplained SCD cases where genetic testing was performed. Follow-up evaluation revealed a clinical diagnosis of an inherited cardiovascular disease was identified in 13% of relatives of SCD victims [7].

The Cardiac Arrest Registry to Enhance Survival (CARES) evaluated out-of-hospital cardiac arrest (OHCA) events of presumed cardiac etiology among individuals who received resuscitative efforts. The registry included 40,274 OHCA records, of which 31,689 OHCA events were presumed to be of cardiac origin (myocardial infarction or arrhythmia) that received prehospital resuscitation. The survival rate to hospital admission was only 26.3%, and the overall survival rate from cardiac arrest to hospital discharge was 9.6% [8].

SCD can have a clear monogenic component (autosomal dominant (AD), autosomal recessive (AR), and sex-linked inheritance) due to the presence of inherited arrhythmia syndromes or cardiomyopathies, particularly in young adults. However, it can also result from polygenic conditions [9]. Mendelian disease variants are typically individually rare in the population; genetic variants associated with inherited cardiac diseases are found in approximately 1% of the general adult population [10]. Such variants are often insufficient on their own to cause a disease phenotype. Common variants may contribute to disease burden or modulate the effects of Mendelian variants. Disorders such as HCM and LQTS are largely Mendelian or near-Mendelian, where variants of large effect sizes can cause or act as protective or regulatory modifiers. In contrast, diseases such as DCM and BrS have complex etiologies involving both non-Mendelian genetic and environmental factors [11]. To date, approximately 4000 genes have been identified as contributing to hereditary cardiomyopathies, arrhythmias, and cardiac conduction disorders [12,13]. Most of the single-nucleotide polymorphisms (SNPs) are located in intergenic regions of the human genome, and affect gene expression and gene regulation, as confirmed by the ENCODE (ENCyclopedia Of DNA Elements) Project [14]. Mutations (MUTs) are very rare, occurring at a frequency of approximately 1 in 1000 individuals. Non-synonymous amino acid exchanges that are neither SNPs nor MUTs are classified as variants of unknown significance (VUS) (0.1–0.5%) as their co-segregation with a particular phenotype has not been established [10]. Systematic investigations of genome-wide SNPs (genome-wide association studies (GWASs)) have primarily focused on SNPs and common cardiovascular diseases (myocardial infarction, arterial hypertension, atrial fibrillation, or quantitative trait loci (QTL) for distinct ECG parameters) [15,16,17,18,19]. GWASs have enabled the identification of numerous genetic variants associated with cardiovascular diseases, leading to the development of genomic or polygenic risk scores (PRSs) that assess an individual’s genetic predisposition to hereditary cardiovascular disorders [20,21,22].

This systematic review aims to identify genetic variants associated with SCD and to emphasize the importance of genetic testing in survivors of sudden cardiac arrest, as well as in the family members of SCD victims for further genetic evaluation.

## 2. Materials and Methods

The review is based on the literature retrieved from the PubMed, Scopus, and Web of Science databases. Searches were conducted using the keywords *sudden cardiac death*, *genetics*, *sudden arrhythmic death*, and *genetic cardiomyopathy*. Inclusion criteria comprised studies published in English that reported data on adults who died from SCD. Exclusion criteria included letters, case reports, and non-peer-reviewed articles. The literature review covered studies published between April 2004 and May 2025, focusing on clinically relevant findings.

The structure of this review is as follows: Section 3 Genetic cardiomyopathies (Section 3.1 Hypertrophic cardiomyopathy, Section 3.2 Dilated cardiomyopathy, and Section 3.3 Arrhythmogenic cardiomyopathy); Section 4 Inherited channelopathies (Section 4.1 Brugada syndrome, Section 4.2 Long QT syndrome, Section 4.3 Short QT syndrome, and Section 4.4 Catecholaminergic polymorphic ventricular tachycardia).

## 3. Genetic Cardiomyopathies

The genetic cardiomyopathies are classified based on morphological and functional features as hypertrophic cardiomyopathy (HCM), dilated cardiomyopathy (DCM), and arrhythmogenic cardiomyopathy (ACM) [23]. Cardiomyocyte-expressed proteins, involved in the pathogenesis of the genetic cardiomyopathies, include sarcomeric, cytoskeletal, desmosomal, and nuclear envelope proteins [24].

### 3.1. Hypertrophic Cardiomyopathy

Hypertrophic cardiomyopathy (HCM) is characterized by asymmetric left ventricular hypertrophy, often with involvement of the interventricular septum. Other variations include apical or mid-wall left ventricular (LV) involvement, accompanied by myocyte hypertrophy and fibrosis [25]. The annual mortality due to SCD alone is low (≤1%) in most HCM patients; however, a subset of patients faces a significantly higher risk (between 4% and 6%). The higher risk groups include patients with ≥15% LV mass showing late gadolinium enhancement (LGE) on magnetic resonance imaging, left ventricular ejection fraction (LVEF) < 50%, LV apical aneurysm, or those with pathogenic sarcomeric mutations, who are candidates for implantable cardioverter-defibrillator (ICD) therapy [1]. Approximately 50–60% of HCM cases are associated with sarcomeric gene mutations, with pathogenic variants in the *MYBPC3* or *MYH7* gene being the most common [26]. Genetic testing primarily includes panel testing for DNA sequence variants in genes with strong evidence of direct involvement in familial HCM (see Table 1), followed by testing for genes with a moderate level of evidence for the development of HCM (see Table 2) (according to ClinGen).

Genetic testing for HCM is also recommended when there is clinical suspicion of syndromic presentations, such as *LAMP2* (Danon disease as phenocopy; genetype caracteristics with environmental impact), *GLA* (Fabry disease), *PRKAG2* (glycogen storage disease), *TTR* (Transthyretin amyloidosis), *GAA* (Pompe disease), and others, as these results can influence therapeutic decisions, for example, aggressive management for Danon disease or enzyme replacement therapy for Fabry disease [33,34,35,36,37]. A likely pathogenic or pathogenic (LP/P) result in a proband warrants cascade genetic testing for first-degree relatives [14]. Carriers of LP/P sarcomere variants (highlighted genes in Table 1) have a poorer prognosis compared to sarcomere variant-negative patients, such as earlier onset of disease, higher incidence of atrial fibrillation and ventricular arrhythmias, heart failure, and higher incidence of SCD [31,37,38,39].

### 3.2. Dilated Cardiomyopathy

Dilated cardiomyopathy (DCM) is defined by left ventricular or biventricular dilatation and systolic dysfunction in the absence of coronary artery disease, sufficient to cause global systolic impairment, or abnormal loading conditions (hypertension; valve disease) [23]. Familial DCM is most often inherited as an autosomal dominant trait, although autosomal recessive, mitochondrial (maternal transmission), or X-linked inheritance can also occur [40,41]. Among patients with newly diagnosed cardiomyopathy, idiopathic DCM is confirmed in approximately 20% to 35% of cases [42]. Genetic testing typically involves panel testing for genes with strong evidence of causing familial DCM (see in Table 3) and, when indicated, genes with moderate evidence (see in Table 4) (according to ClinGen).

*LMNA*, *RBM20*, and *FLNC* gene mutations are associated with an increased risk of ventricular arrhythmias and SCD [43,44,45]. Desmosomal and *LMNA* variants show a particularly strong association with SCD and ventricular arrhythmias, independent of LVEF (left ventricular ejection fraction) [46]. Truncating variants in the titin gene (*TTN*) are the most frequent, occurring in approximately 20% of DCM cases [47]. Some DCM-mutated genes, e.g., genes coding desmosomal proteins and ion flux, overlap with other cardiomyopathy subtypes, such as ACM, but also with channelopathies [13]. Pathogenic variants can be identified in up to 50% of DCM patients [13]. A complex polygenic inheritance pattern with environmental influences may contribute to disease manifestation [48,49].

Genetic testing is recommended for DCM patients with the highest yield of pathogenic variant screening and should be considered even in the absence of familial context or associated clinical features (<60 years of age). The most common genes associated with DCM (*TTN*, *LMNA*, and *FLNC*) should be included in the panel [14]. An LP/P result in the proband warrants cascade genetic testing of first-degree relatives [14]. Early ICD implantation should be considered in *LMNA* carriers with truncating mutations, while those with missense variants have lower arrhythmic risk. A higher risk of SCD is also associated with pathogenic variants (truncated variants) in *FLNC*, *DES*, *RBM20*, and *PLN* genes [14,50,51]. According to the 2022 ESC guidelines, ICD implantation should be considered in DCM patients with a pathogenic *LMNA* variant when the estimated 5-year risk of life-threatening ventricular arrhythmia (VA) is ≥10% or in those with LVEF < 50%, non-sustained (NSVT), or atrioventricular (AV) conduction delay. ICD therapy is also recommended in patients with a LVEF < 50% and ≥2 risk factors (syncope, LGE on cardiac MR, inducible SMVT at PES, or pathogenic variants in *LMNA*, *PLN*, FLNC, and *RBM20* genes) [1]. In 2019, the “Risk Prediction Score for Life-Threatening Ventricular Tachyarrhythmias in Laminopathies” was introduced. This model was developed to estimate the 5-year risk of life-threatening ventricular tachyarrhythmias (VTAs) in individuals carrying LMNA mutations. In this study, the main predictors of LTVAs, defined as either SCD or ICD-treated hemodynamically unstable ventricular tachycardia, included the following:

(1) Male sex;
(2) Presence of non-missense LMNA mutations;
(3) First-degree or higher atrioventricular block;
(4) Episodes of non-sustained ventricular tachycardia;
(5) Reduced left ventricular ejection fraction [52].

In the REDLAMINA registry cohort, which included 140 carriers of pathogenic LMNA variants, the only independent predictors of major arrhythmic events were non-sustained ventricular tachycardia (NSVT) and a left ventricular ejection fraction (LVEF) below 45% [46].

### 3.3. Arrhythmogenic Cardiomyopathy

Arrhythmogenic cardiomyopathy (ACM), also referred to as arrhythmogenic right ventricular cardiomyopathy (ARVC), is an inherited heart muscle disorder characterized by progressive fibro and fibro-fatty myocardial replacement, which serves as a substrate for ventricular arrhythmias and SCD. The classic ARVC phenotype primarily affects the right ventricle (RV), but biventricular (BiVACM), right-dominant (ARVC), and left-dominant (ALVC) forms also occur [53]. ACM is most commonly inherited as an autosomal dominant trait; however, syndromic forms such as Naxos disease and Carvajal syndrome follow an autosomal recessive pattern [54]. Mutations in five genes encoding desmosomal proteins, desmoplakin (*DSP*), plakophillin (*PKP2*), plakoglobin (*JUP*), desmoglein-2 (*DSG2*), and desmocollin-2 (*DSC2*), are identified in up to 50% of patients. Several non-desmosomal genes have also been implicated, though their pathogenic roles are less well established [55,56] (Table 5 and Table 6).

Cohort studies have reported that 4–16% of ACM patients carry multiple desmosomal gene mutations. Individuals with >1 desmosomal variant have an increased risk of arrhythmias and SCD [57,58].

Genetic testing identifies pathogenic variants in approximately two-thirds of ACM cases [58]. Disease penetrance among first-degree relatives ranges from 28 to 58% [59,60].

The incidence and predictors of ICD therapy in patients with ARVD were investigated in a cohort on 86 patients after receiving ICDs for primary prevention. Appropriate ICD therapy was delivered in 48% of patients during follow-up [61]. ICD implantation should therefore be considered in primary prevention patients with definite ARVC and an arrhythmic syncope, or severe RV or LV systolic dysfunction, moderate right or left ventricular dysfunction, or previously documented NSVT [1]. Genetic testing is recommended for all patients with phenotypic features of ACM, with reference to definitive disease-associated genes (currently *PKP2*, *DSP*, *DSG2*, *DSC2*, *TMEM43*, *PLN*, *FLNC*, *DES*, and *LMNA*), and variant-specific genetic testing is recommended for family members [14].

The 2019 ARVC risk calculator estimates the 5-year risk of ventricular arrhythmias (VAs) in patients with a definite diagnosis of arrhythmogenic cardiomyopathy (ACM). The calculator incorporates several clinical criteria, including age at diagnosis, sex, recent cardiac syncope (<6 months), number of inverted T waves on a 12-lead ECG, maximum number of premature ventricular contractions (PVCs) on 24 h Holter monitoring, history of non-sustained ventricular tachycardia (NSVT), and right ventricular ejection fraction (RVEF). A cohort study of 544 patients applied the ARVC risk model to compare four genetic groups: PKP2, desmoplakin, other desmosomal genes, and gene-elusive cases. The study found that the model performed best in carriers of PKP2 variants compared with gene-negative individuals [62].

The desmoplakin (DSP) risk score is a clinical model designed to individualize the assessment of VA risk in patients carrying pathogenic DSP variants. Independent predictors of VA included in the score are female sex, history of NSVT, the natural logarithm of PVCs burden on 24 h Holter monitoring, left ventricular ejection fraction (LVEF) below 50%, and moderate to severe right ventricular systolic dysfunction. Based on these parameters, patients are stratified into three risk categories: low (0–5% risk at 5 years), intermediate (5–20%), and high (>20%) [63].

## 4. Inherited Channelopathies

Inherited cardiac channelopathies arise from mutations in genes encoding cellular components that regulate calcium ion availability or membrane ion channels, including those for sodium, potassium, or calcium [64]. The coordinated transmembrane transport of these ions in cardiac myocytes is critical for maintaining normal cardiac electrophysiology and rhythm. Pathogenic variants in genes encoding these ion channels disrupt ionic conduction, predisposing to channelopathies and ventricular arrhythmias, which may culminate in sudden cardiac death (SCD) [64]. The principal hereditary channelopathies comprise long QT syndrome (LQTS), short QT syndrome (SQTS), Brugada syndrome (BrS), and catecholaminergic polymorphic ventricular tachycardia (CPVT), all of which are often characterized by distinct ECG abnormalities that may manifest at baseline or under specific conditions such as physical exertion (CPVT and LQTS), fever (BrS), or pharmacological provocation (BrS) [64,65,66].

### 4.1. Brugada Syndrome

Brugada syndrome is an inherited arrhythmogenic disorder characterized by distinctive electrocardiographic pattern (ST-segment elevation in the right precordial leads) associated with malignant ventricular arrhythmias (ventricular fibrillation) and an increased risk of SCD, infrequently with conduction disorders and atrial arrhythmias [1,14]. Brugada syndrome (BrS) type I electrocardiographic morphology is defined by ST-segment elevation ≥ 2mm in at least one lead of the right precordial leads (V_1_–V_2_) positioned in the 4th intercostal space, occurring either spontaneously or following pharmacological provocation with a class I antiarrhythmic agent [14]. Multiple isoforms of the cardiac sodium channel have been identified (INa 1.1–1.9); among these, isoform NaV1.5 has been isolated in the human heart and consists of an α and an auxiliary β-subunit [63]. The α-subunit encoded by the SCN5A gene is sufficient to form a functional sodium channel, whereas the β-subunits modulate channel expression levels and influence voltage-dependent inactivation. Pathogenic variants in sodium channel genes may reduce channel expression, accelerate inactivation, or cause incomplete inactivation during sustained depolarization. These alterations promote a proarrhythmic substrate and are implicated in the pathogenesis of long QT syndrome (LQTS), Brugada syndrome (BrS), and various cardiac conduction disorders [67,68]. Rare pathogenic variants in *SCN5A* (locus 3p22.2; loss of INa1.5 channel function) are identified in approximately 15–30% of Brugada syndrome (BrS) cases and are the only definite genetic variants causing the disease. (ClinGen.) In a study involving 13 large families comprising 115 mutation carriers, the role of an *SCN5A* mutation in causing a BrS phenotype was investigated and proved that there was incomplete penetrance; asymptomatic carriers of the *SCN5A* variant can show a positive provocative drug challenge [69].

Tadros et al. investigated genetic determinants of ajmaline-induced PR and QRS interval changes and their association with type I BrS. The study demonstrated that genetic factors that affect sodium channel function, in combination with polygenic risk scores for Brugada syndrome (PRSBrS) derived from genome-wide association studies, family history, and baseline electrocardiographic parameters, can predict the development of a diagnostic, drug-induced type I BrS pattern [70]. Implantable cardioverter-defibrillator (ICD) implantation is recommended in patients with BrS who have survived cardiac arrest or experienced documented malignant ventricular arrhythmias or arrhythmic syncope, with strict avoidance of precipitating factors [1]. The presence of an LP/P *SCN5A* variant supports the diagnosis of BrS in patients with a type I BrS electrocardiogram (ECG) pattern; however, its absence does not exclude the diagnosis [14]. Genetic testing of all first-degree relatives of affected individuals is recommended to facilitate cascade screening and early risk stratification [71].

### 4.2. Long QT Syndrome

Congenital long QT syndrome (LQTS) is a hereditary channelopathy characterized by dysfunction in cardiac repolarization. It is typically identified on baseline electrocardiogram (ECG) by QT interval prolongation (QTc ≥ 480 ms or an LQTS risk score > 3), often accompanied by T-wave abnormalities, such as biphasic or notched T waves [1,72,73,74]. Genetic testing for LQTS primarily involves panel testing for genes with strong and definitive evidence of causality in familial LQTS (see Table 7) and a gene with a moderate level of evidence for the development of the disease, *CACNA1C* (locus 12p13.3; L-type calcium channel) (ClinGen).

Genetic testing can identify mutations in approximately 75% of patients with long QT syndrome (LQTS), with 90% of positive genotypes involving genes with definitive evidence of causality for the disease [55]. LQTS subtypes are categorized based on the mode of inheritance:

(1) Autosomal-dominant LQTS without extra-cardiac manifestation.
(2) Autosomal-dominant LQTS with extra-cardiac manifestation, including Andersen–Tawil syndrome (LQT7), characterized by frequent ventricular arrhythmias, facial dysmorphology, and periodic paralysis, and Timothy Syndrome (LQT8), characterized by prolonged QT, cardiac malformations, syndactyly, autism spectrum disorder, and dysmorphism.
(3) Autosomal recessive LQTS, including Jervell and Lange–Nielsen Syndrome, which combines extreme QT prolongation with congenital deafness [75,76,77].

Based on their mechanisms of action, LQTS-associated genes can be classified into the following groups:

(1) Genes that reduce the outward potassium current: *KCNQ1* and *KCNH2* account for approximately 80% of genetically confirmed LQTS cases, as well as *KCNE1* and *KCNE2* are associated with milder phenotypes.
(2) Genes that increase inward sodium current: *SCN5A*, which can cause overlapping syndromes such as LQTS, Brugada syndrome, and cardiac conduction abnormalities.
(3) Genes that increase inward calcium current: *CALM1*, *CALM2*, and *CALM3* [78,79,80].

Mutations in *CALM* genes are associated with a high incidence of malignant arrhythmias. According to the International Calmodulinopathy Registry, life-threatening arrhythmias were observed in 78% of cases, with a mean QTc interval of nearly 600 ms [81]. Genotype–phenotype studies have shown that, in most cases, LQTS results from loss-of-function mutations in *KCNQ1* (LQTS Type 1—LQT1) and *KCNH2* (LQTS Type 2—LQT2) or gain of function in *SCN5A* mutations (LQTS Type 3—LQT3), all of which predispose young, otherwise healthy individuals to life-threatening arrhythmias [82,83,84,85,86].

Mazzanti et al. present the 1-2-3-LQTS-Risk model, a validated 5-year risk score for patients with long QT syndrome (LQTS), and recommend ICD implantation for those with a 5-year risk ≥ 5% (number needed to treat [NNT] = 9) [86]. The electrocardiographic (ECG) characteristics of LQTS subtypes include the following:

(1) LQT1: Broad-based T waves, with cardiac events typically triggered by exercise.
(2) LQT2: Low-amplitude or notched T waves, with arrhythmias often triggered by auditory stimuli.
(3) LQT3: A long isoelectric ST-segment, with cardiac events commonly occurring during sleep.

LQT1 patients have a better response to β blockers; LQT2 and LQT3 are associated with a more malignant form of the disease [1,87]. Cascade screening of family members of patients with LQTS is recommended, even if they do not meet ECG criteria [87].

Molecular genetic testing for disease-associated genes is recommended for all patients who meet one of the following criteria: acquired long QT syndrome (LQTS) with a history of drug-induced torsades de pointes (TdP), or individuals under 40 years of age with a QTc > 440 ms in men or >450 ms in women [14]. Gene-specific management of LQTS has become increasingly feasible in clinical practice. For example, patients with the *KCNQ1* variant are at higher risk during sympathetic stimulation and can benefit from antiadrenergic intervention, such as betablockers and left cardiac sympathetic denervation (LCSD). In patients with *KCNH2*-associated LQTS, maintaining adequate potassium levels is crucial. Additionally, mexiletine treatment has been shown to be effective in patients with *SCN5A* and *KCNH2* variants [78,88,89,90,91,92,93].

### 4.3. Short QT Syndrome

Short QT syndrome (SQTS) is a channelopathy characterized by a short QT interval on the baseline electrocardiogram (ECG), which increases the risk of atrial fibrillation and ventricular arrhythmias. The diagnostic criteria for SQTS include a QTc ≤ 320 ms alone, or a QTc ≤ 360 ms in combination with a family history of SQTS, or aborted cardiac arrest in the absence of structural heart disease, or the presence of a pathogenic mutation [1]. Genetic testing for SQTS primarily involves panel testing for genes with definitive and strong evidence of causality, including *KCNH2* (locus 7q35-36; increase in IKr channel function) and *KCNQ1* (locus 11p15.5; increase in IKs channel function). Genes with moderate evidence for SQTS include *KCNJ2* (locus 17q23; increase in IK1 channel function) and *SLC4A3* (locus 2q35) (according to ClinGen). Autosomal recessive primary systemic carnitine deficiency syndrome is characterized by progressive cardiomyopathy, skeletal myopathy, hypoglycemia and hyperammonemia and is caused by variants in *SLC22A5*. It is also associated with SQTS and SCD and studies have shown that the QT interval in this condition is responsive to carnitine supplementation [92]. Quinidine currently provides the best results in treating SQTS and QT interval prolongation [1]. ICD implantation should also be considered for SQTS patients who have experienced a cardiac arrest event or have documented spontaneous sustained ventricular tachycardia (VT). ICD implantation should be considered in SQTS patients with arrhythmic syncope [1]. Variant-specific genetic testing for family members of patients with SQTS is recommended [14].

### 4.4. Catecholaminergic Polymorphic Ventricular Tachycardia

Catecholaminergic polymorphic ventricular tachycardia (CPVT) is a rare, malignant heritable arrhythmia syndrome characterized by polymorphic or bidirectional ventricular tachycardia in young individuals, typically triggered by physical or emotional stress. Affected individuals may have a normal baseline electrocardiogram (ECG), and diagnosis is based on the occurrence of arrhythmia during exercise stress test or Holter ECG recording [1,14,94,95]. Genetic testing primarily includes panel testing for genes with definitive and strong evidence for CPVT (see Table 8) as well as genes with moderate evidence, such as *CALM 1–3* (locus 14q32.11, 2p21, and 19q13.32), which affect *RyR2* binding affinity and lead to inappropriate Ca^2+^ release from the sarcoplasmic reticulum (SR).

The *RyR2* gene, which encodes the sarcoplasmic reticulum (SR) Ca^2+^ channel known as *RyR*, is associated with an autosomal dominant inheritance pattern and is found in nearly 60% of patients with CPVT [96,97,98,99]. Mutations in the CASQ2 gene, which encodes calsequestrin-2 (a protein that binds free calcium in the SR), are less common and are typically inherited in an autosomal recessive manner [99]. Mutations in the *KCNJ2* gene, which are also implicated in Andersen–Tawil syndrome have been described in a few CPVT patients with a normal QTc interval but a CPVT phenotype [100]. For phenotype-positive CPVT patients who test negative for known CPVT associated genes, genetic testing may be considered for CPVT phenocopies caused by pathogenic variants in the *KCNJ2*, *SCN5A*, and *PKP2* genes [101,102]. Variant-specific genetic testing is recommended for family members of CPVT patients to identify the disease-causative variant [14]. While ICD implantation is certainly indicated for secondary prevention of sudden cardiac death, some studies, including a systematic review of 53 studies, have raised concerns about potential harmful consequences of ICD implantation for primary prevention. For example, a fatal ICD-related electrical storm (4/1429 patients) and several other complications unrelated to frequent device activation have also been reported [103].

## 5. Discussion

Genetic cardiomyopathies represent a spectrum of inherited myocardial disorders in which pathogenic variants drive disease mechanisms, influence prognosis, and enable timely interventions. In individuals with HCM, DCM, or ACM, family genotype screening not only identifies at-risk relatives early but also guides targeted ICD therapy [23,24] (Table 9).

A total of 50–60% of familial HCM cases harbor sarcomeric mutations (most commonly MYBPC3 and MYH7) [26]. Carriers of P/LP variants have a more severe clinical course, earlier disease onset, higher arrhythmic burden, progression to heart failure, and increased risk of SCD [37,38,39]. Significant risk markers include left ventricular ejection fraction (LVEF) < 50%, apical aneurysm, or extensive LGE, with the presence of sarcomeric mutations, which identify patients most likely to benefit from prophylactic ICD implantation [1].

In approximately 50% of DCM patients, pathogenic gene variants (TTN, LMNA, and FLNC) are identified [46,47]. Autosomal dominant inheritance is the most common, although recessive, X-linked, and mitochondrial patterns have also been reported [40,41]. LMNA and FLNC mutations are strongly associated with an increased risk of malignant VA and SCD [14,51,52]. Clinical tools, e.g., LMNA Risk Prediction Score, enable individual estimation of malignant arrhytmias. Current guidelines recommend early ICD consideration in LMNA mutation carriers with an estimated 5-year risk of life-threatening VA ≥ 10% or in those with an LVEF < 50%, NSVT, or AV conduction delay [1]. These data highlight the critical role of genotype-informed frameworks in guiding prognosis and individualized management strategies for DCM.

Mutations in desmosomal genes—including PKP2, DSP, DSG2, DSC2, and TMEM43—cause up to half of cases of ACM, while non-desmosomal genes such as PLN, FLNC, DES, and LMNA contribute additional etiologic pathways [57,58]. Individuals with multiple desmosomal gene mutations exhibit a phenotype characterized by higher VA burden and increased SCD risk [57,58]. Approximately half of patients with definite ACM diagnosis receiving ICDs (primary prevention) will experience appropriate therapy during follow-up [62]. Risk estimation tools, e.g., 2019 ARVC Risk Calculator and the DSP-specific arrhythmic risk score, offer gene and phenotype-associated prediction of VA, enabling more precise indication for ICD implantation [62].

Inherited channelopathies represent a group of genetically mediated arrhythmogenic disorders in which pathogenic variants alter transmembrane transport of sodium, potassium, and calcium ions, predisposing VA and SCD. The major phenotypes demonstrate genotype–phenotype correlations that are critical for risk stratification and management.

Brugada syndrome is predominantly associated with loss-of-function variants in SCN5A, although penetrance is incomplete and additional polygenic modifiers influence phenotypic expression. Electrocardiographic manifestations may be spontaneous or pharmacologically provoked, with ICD implantation reserved for survivors of cardiac arrest, documented malignant arrhythmias, or arrhythmic syncope. Family cascade screening and genotype-informed risk assessment remain central to early detection of at-risk relatives.

Long QT syndrome is primarly associated with KCNQ1, KCNH2, and SCN5A gene mutations [79,80,81]. Mechanistic classification based on altered potassium, sodium, or calcium current alterations enables individualized therapy (β-blockade, mexiletine, left cardiac sympathetic denervation, and ICD implantation in high-risk patients) [93,94,95,96,97,98,99]. Gene-specific management, e.g., LQTS caused by KCNQ1 mutation, illustrates the potential for precision medicine to mitigate arrhythmic risk [83,84,85,86,87].

SQTS and CPVT demonstrate the heterogeneity and phenotypic complexity of channelopathies [92,96]. SQTS is caused by gain-of-function potassium channel mutations, predisposing patients to VA, whereas CPVT is associated with dysregulated sarcoplasmic reticulum calcium handling (RyR2 and CASQ2 variants) [92]. Genetic testing facilitates identification of patients with both conditions and adequate pharmacologic therapy and ICD implantation.

## 6. Future Directions

Further research is necessary to clarify genotype–phenotype correlations, particularly for genes with moderate evidence of pathogenicity. Clinical risk models for genetically determined cardiac disorders with a high incidence of malignant arrhythmias are valuable in guiding timely therapeutic interventions, including antiarrhythmic medications and ICD implantation. As genetic testing becomes more widely accessible, incorporating genetic analyses into the evaluation of patients with hereditary cardiomyopathies and channelopathies is expected to optimize therapy, enhance quality of life, and improve long-term outcomes in these patients.

## 7. Conclusions

This review consolidates evidence linking specific genetic variants to an elevated risk of sudden cardiac death (SCD). The findings underscore the pivotal role of comprehensive genetic screening and early clinical intervention in individuals with a family history of SCD or with risk factors for inherited cardiac disorders predisposing to SCD. Systematic implementation of genetic cascade testing within affected families remains essential to identify at-risk relatives. Furthermore, gene-specific management of familial cardiomyopathies and inherited cardiac channelopathies should be prioritized to facilitate proactive family screening, enable timely preventive strategies, and inform precision-based therapeutic approaches. Continued research in this field is critical to advance targeted treatments and improve clinical outcomes for individuals with these heritable cardiac disorders.

## Figures and Tables

**Table 1:** Genes with strong evidence for direct involvement in causing familial HCM [27,28,29,30,31,32].

Gene	Specifics	Inheritance Pattern
***MYBPC3***	locus 11p11.2, myosin-binding protein C3	AD
***MYH7***	locus 14q11.2-q12, beta myosin heavy chain	AD
***TNNI3***	locus 19q13.49, troponin I, cardiac type	AD
***TNNC1***	locus 3p21.1, troponin C, cardiac type	AD
*TPM1*	locus 15q22.2, alpha tropomyosin	AD
***ACTC1***	locus 15q.14, alpha cardiac actin	AD
***MYL2***	locus 12q24.11, regulatory myosin light chain	AD
***MYL3***	locus 3p21.31, essential myosin light chain	AD
*PM1*	locus 15q22.2, alpha tropomyosin	AD
*FHOD3*	locus 18q12.2, formin homology 2 domain containing 3	AD
*FLNC*	locus 7q32.1, filamin C	AD
*ALPK3*	locus 15q25.3, alpha kinase 3	AR
*PRKAG2*	locus 7q36.1, protein kinase AMP-activated non-catalytic subunit γ 2	AD
*CACNA1C*	locus 12p13.33, calcium voltage-gated channel subunit alpha1 C	AD
*ACTN2*	locus 1q43, actinin alpha 2	AD
*CSRP3*	locus 11p15.1, cysteine- and glycine-rich protein 3	AD, AR
***TNNT2***	locus 1q32.1, troponin T, cardiac type	AD

Legend: AD (for autosomal dominant); AR (for autosomal recessive).

**Table 2:** Genes with moderate level of evidence for direct involvement in causing familial HCM. (according to ClinGen).

Gene	Specifics	Inheritance Pattern
*KLHL24*	locus 3q27.1, Kelch-like family member	AR
*TRIM63*	locus 1p36.11, tripartite motif containing	AR
*JPH2*	locus 20q13.12, junctophilin	AD

Legend: AD (for autosomal dominant); AR (for autosomal recessive).

**Table 3:** Genes with strong evidence of involvement in familial DCM (ClinGen).

Gene	Specifics	Inheritance Pattern
*BAG3*	locus 10q26.11, myopathy BAG family molecular chaperone regulator 3	AD
*DES*	locus 2q35, desmin	AD
*FLNC*	locus 7q32.1, filamin-C	AD
*LMNA*	locus 1q22, lamin A/C	AD
*MYH7*	locus 14q11.2, β myosin heavy chain	AD
*RBM20*	locus 10q25.2 RNA-binding motif protein 20	AD
*SCN5A*	locus 3p22.2, sodium channel protein type 5 subunit alpha	AD
*TNNC1*	locus 3p21.1, cardiac troponin C	AD
*TTN*	locus 2q31.2, titin	AD
*TNNT2*	locus 1q32.1, troponin T	AD
*JPH2*	locus 20q13.12, junctophilin 2	AR
*TNNI3*	locus 19q13.4, cardiac troponin I	AD
*VCL*	locus 10q22.2, metavinculin	AD
*LDB3*		AR
*NRAP*		AR
*PPA2*		AR

Legend: AD (for autosomal dominant); AR (for autosomal recessive).

**Table 4:** Genes with moderate evidence for involvement in familial DCM. (ClinGen).

Gene	Specifics	Inheritance Pattern
*ACTC1*	locus 15q11q14, cardiac alpha-actin	AD
*BAG5*	myopathy BAG family molecular chaperone regulator 3	AR
*RPL3L*	ribosomal protein L3-like	AR
*FLII*		AR
*MYLK3*		AD
*MYZAP*		AR

Legend: AD (for autosomal dominant); AR (for autosomal recessive).

**Table 5:** Genes most strongly associated with ACM (ClinGen).

Gene	Specifics	Phenotype of ACM	Inheritance Pattern
*DSC2*	locus 18q12.1, desmocollin 2 (desmosome)	ARVC, less frequent BiVACM and ALVC	AD
*DSG2*	locus 18q12.1, desmoglein 2 (desmosome)	BiVACM and ALVC	AD
*DSP*	locus 6p24.3, desmoplakin (desmosome)	BiVACM and ALVC	AD
*PKP2*	locus 12p11.21, plakophilin 2 (desmosome)	ARVC, less frequent BiVACM and ALVC	AD
*TMEM43*	locus 3p25.1, transmembrane protein 43 (nuclear envelope)	ARVC and BiVACM	AD

Legend: AD (for autosomal dominant); AR (for autosomal recessive).

**Table 6:** Genes with moderate evidence for ACM (ClinGen).

Gene	Specifics	Phenotype of ACM	Inheritance Pattern
*PLN*	locus 6q22.31, phospholamban (sarcoplasmic reticulum; calcium handling)	ALVC/DCM	AD
*DES*	locus 2q35, desmin (cytoskeleton)	ALVC with conduction system abnormalities	AD

Legend: AD (for autosomal dominant); AR (for autosomal recessive).

**Table 7:** Genes with definitive and strong evidence of causing familial LQTS.

Gene	Specifics	Inheritance Pattern
*CALM1*	locus 14q32.11, L-type calcium channel	AD
*CALM2*	locus 2p21, L-type calcium channel	AD
*CALM3*	locus 19q13.32, L-type calcium channel	AD
*KCNQ1*	locus 11p15.5, loss-of-IKs channel function 40–55%	AD
*KCNH2*	locus 7q35-36, loss-of-IKr channel function 30–45%	AD
*SCN5A*	locus 3p21-p24, increase in INa1.5 channel function 5–10%	AD
*TRDN*	locus 6q22.31, L-type calcium channel	AR

Legend: AD (for autosomal dominant); AR (for autosomal recessive).

**Table 8:** Genes with definitive and strong evidence of causing CPVT.

Gene	Specifics	Inheritance Pattern
*RyR2*	locus 1q43, inappropriate Ca^2+^ release from the SR	AD
*CASQ2*	locus 1p13.1, inappropriate Ca^2+^ release from the SR	AR
*TECRLa*	locus 4q13.1, altered Ca^2+^ homeostasis, possibly linked to fatty acid/lipid metabolism	AR
*TRDNa*	locus 6q22.31, expression leading to remodeling of the cardiac dyad/calcium release unit	AR
*KCNJ2*	locus 17q24.3, loss-of-IK1 channel function	AD

Legend: AD (for autosomal dominant); AR (for autosomal recessive).

**Table 9:** Inheritance patterns of genetically associated cardiac conditions.

Genetically Associated Cardiac Condition	Inheritance Pattern
hypertrophic cardiomyopathy (HCM)	**AD**, AR, and X-linked
dilated cardiomyopathy (DCM)	**AD**, AR, X-linked, and mitochondrial
arrhythmogenic cardiomyopathy (ACM)	**AD**, AR
long QT syndrome (LQTS)	**AD**, AR
short QT syndrome (SQTS)	**AD**, AR, and sporadic cases
Brugada syndrome (BrS)	**AD**, AR, X-linked, de novo mutations, and polygenic pattern
catecholaminergic polymorphic ventricular tachycardia (CPVT)	**AD**, AR

Legend: AD (for autosomal dominant); AR (for autosomal recessive). Bold letters indicate the most common inheritance pattern; non-bold letters indicate rarer forms.

## Data Availability

Not applicable.

## Abbreviations

The following abbreviations are used in this manuscript:

ACM	arrhythmogenic cardiomyopathy
ACTC1	alpha cardiac actin
ACTN2	actinin alpha 2
AD	autosomal dominant
ALPK3	alpha kinase 3
ALVC	dominant-left arrhythmogenic cardiomyopathy
ARIC	Atherosclerosis Risk in Communities Study
ARVC	arrhythmogenic right ventricular cardiomyopathy
ARVC	dominant-right arrhythmogenic cardiomyopathy
AR	autosomal recessive
BrS	Brugada syndrome
Bi-VACM	biventricular disease variants
CACNA1C	calcium voltage-gated channel subunit alpha1 C
CALM1, CALM2, CALM3	calmodulin 1-3
CARES	Cardiac Arrest Registry to Enhance Survival
CPVT	catecholaminergic polymorphic ventricular tachycardia
CSRP3	cysteine- and glycine-rich protein 3
DCM	dilated cardiomyopathy
DES	desmin
DSC2	desmocollin-2
DSG2	desmoglein-2
DSP	desmoplakin
ECG	electrocardiogram
ENCODE	ENCyclopedia Of DNA Elements
FHOD3	formin homology 2 domain containing 3
FLNC	filamin C
GWASs	genome-wide association studies
HCM	hypertrophic cardiomyopathy
ICD	cardioverter defibrillator
JPH2	junctophilin
JUP	plakoglobin
KLHL24	Kelch-like family member
LCSD	left cardiac sympathetic denervation
LGE	late gadolinium enhancement
LMNA	lamin A/C
LP/P	likely pathogenic or pathogenic
LV	left ventricular
LVEF	left ventricular ejection fraction
LQTS	long QT syndrome
MR	magnetic resonance
MUT	mutations
MYBPC3	myosin-binding protein C3
MYH7	beta myosin heavy chain
MYL2	regulatory myosin light chain
MYL3	essential myosin light chain
NSVT	non-sustained ventricular tachycardia
OHCA	out-of-hospital cardiac arrest
PKP2	plakophillin
PLN	phospholamban
PM1	alpha tropomyosin
PRSBrS	polygenic risk scores for Brugada syndrome
PES	Programmed Electrical Stimulation
PRKAG2	protein kinase AMP-activated non-catalytic subunit gamma 2
PRS	polygenic risk scores
PVCs	premature ventricular contractions
QTL	quantitative trait loci
RBM20	RNA-binding motif protein 20
RPL3L	ribosomal protein L3-like
RV	right ventricular
RyR	Ryanodine
RVEF	right ventricular ejection fraction
SCD	sudden cardiac death
SCN5A	sodium channel protein type 5 subunit alpha
SNP	single-nucleotide polymorphism
SQTS	short QT syndrome
SMVT	sustained monomorphic ventricular tachycardia
TdP	Torsades de Pointes
TMEM43	transmembrane protein 43
TNNC1	troponin C, cardiac type
TNNI3	troponin I, cardiac type
TPM1	alpha tropomyosin
TRIM63	tripartite motif containing
VCL	metavinculin

## References

[1] Zeppenfeld K., Tfelt-Hansen J., De Riva M., Winkel B.G., Behr E.R., Blom N.A., Philippe F., Corrado D., Dagres N., de Chillou C. (2022). ESC Guidelines for the management of patients with ventricular arrhythmias and the prevention of sudden cardiac death. Eur. Heart J..

[2] Bidzimou M.K., Landstrom A.P. (2022). From diagnostic testing to precision medicine: The evolving role of genomics in cardiac channelopathies and cardiomyopathies in children. Curr. Opin. Genet. Dev..

[3] Krahn A.D., Connolly S.J., Roberts R.S., Gent M., ATMA Investigators (2004). Diminishing proportional risk of sudden death with advancing age: Implications for prevention of sudden death. Am. Heart J..

[4] Bougouin W., Lamhaut L., Marijon E., Jost D., Dumas F., Deye N., Beganton F., Empana J.P., Chazelle E., Cariou A. (2014). Characteristics and prognosis of sudden cardiac death in Greater Paris: Population-based approach from the Paris Sudden Death Expertise Center (Paris-SDEC). Intensive Care Med..

[5] Agesen F.N., Lynge T.H., Blanche P., Banner J., Prescott E., Jabbari R., Hansen J.T. (2021). Temporal trends and sex differences in sudden cardiac death in the Copenhagen City Heart Study. Heart.

[6] Zhao D., Post W.S., Blasco-Colmenares E., Cheng A., Zhang Y., Deo R., Pastor-Barriuso R., Michos E.D., Sotoodehnia N., Guallar E. (2019). Racial differences in sudden cardiac death. Circulation.

[7] Bagnall R.D., Weintraub R.G., Ingles J., Duflou J., Yeates L., Lam L., Davis A.M., Thompson T., Connell V., Wallace J. (2016). A Prospective Study of Sudden Cardiac Death among Children and Young Adults. N. Engl. J. Med..

[8] McNally B., Robb R., Mehta M., Vellano K., Valderrama A.L., Yoon P.W., Sasson C., Crouch A., Perez A.B., Merritt R. (2011). Out-of-Hospital Cardiac Arrest Surveillance: Cardiac Arrest Registry to Enhance Survival (CARES), United States, October 1, 2005–December 31, 2010.

[9] Khera A.V., Mason-Suares H., Brockman D., Wang M., VanDenburgh M.J., Senol-Cosar O., Patterson C., Newton-Cheh C., Zekavat S.M., Pester J. (2019). Rare Genetic Variants Associated With Sudden Cardiac Death in Adults. J. Am. Coll. Cardiol..

[10] Stallmeyer B., Schulze-Bahr E. (2015). Cardiovascular disease and sudden cardiac death: Between genetics and genomics. Eur. Heart J..

[11] Walsh R., Tadros R., Bezzina C.R. (2020). When genetic burden reaches threshold. Eur. Heart J..

[12] Claussnitzer M., Cho J.H., Collins R., Cox N.J., Dermitzakis E.T., Hurles M.E., Kathiresan S., Kenny E.E., Lindgren C.M., MacArthur D.G. (2020). A brief history of human disease genetics. Nature.

[13] Roberts R., Marian A.J., Dandona S., Stewart A.F. (2013). Genomics in cardiovascular disease. J. Am. Coll. Cardiol..

[14] Gerstein M.B., Kundaje A., Hariharan M., Landt S.G., Yan K.K., Cheng C., Mu X.J., Khurana E., Rozowsky J., Alexander R. (2012). Architecture of the human regulatory network derived from ENCODE data. Nature.

[15] Schunkert H., Konig I.R., Kathiresan S., Reilly M.P., Assimes T.L., Holm H., Preuss M., Stewart A.F.R., Barbalic M., Gieger C. (2011). Large-scale association analysis identifies 13 new susceptibility loci for coronary artery disease. Nat. Genet..

[16] Sabater-Lleal M., Huang J., Chasman D., Naitza S., Dehghan A., Johnson A.D., Teumer A., Reiner A.P., Folkersen L., Basu S. (2013). Multiethnic meta-analysis of genome-wide association studies in >100,000 subjects identifies 23 fibrinogen-associated loci but no strong evidence of a causal association between circulating fibrinogen and cardiovascular disease. Circulation.

[17] Ehret G.B., Munroe P.B., Rice K.M., Bochud M., Johnson A.D., Chasman D.I., Smith A.V., Tobin M.D., Verwoert G.C., Hwang S.J. (2011). Genetic variants in novel pathways influence blood pressure and cardiovascular disease risk. Nature.

[18] Ritchie M.D., Denny J.C., Zuvich R.L., Crawford D.C., Schildcrout J.S., Bastarache L., Ramirez A.H., Mosley J.D., Pulley J.M., Basford M.A. (2013). Genome- and phenome-wide analyses of cardiac conduction identifies markers of arrhythmia risk. Circulation.

[19] Arking D.E., Pulit S.L., Crotti L., van der Harst P., Munroe P.B., Koopmann T.T., Sotoodehnia N., Rossin E.J., Morley M., Wang X. (2014). Genetic association study of QT interval highlights role for calcium signaling pathways in myocardial repolarization. Nat. Genet..

[20] Bezzina C.R., Barc J., Mizusawa Y., Remme C.A., Gourraud J.B., Simonet F., Verkerk A.O., Schwartz P.J., Crotti L., Dagradi F. (2013). Common variants at SCN5A-SCN10A and HEY2 are associated with Brugada syndrome, a rare disease with high risk of sudden cardiac death. Nat. Genet..

[21] Turkowski K.L., Dotzler S.M., Tester D.J., Giudicessi J.R., Bos J.M., Speziale A.D., Vollenweider J.M., Ackerman M.J., Cheng S., Funke B.H. (2020). Corrected QT interval-polygenic risk score and its contribution to type 1, type 2, and type 3 long-QT syndrome in probands and genotype-positive family members. Circ. Genom. Precis. Med..

[22] Wijeyeratne Y.D., Tanck M.W., Mizusawa Y., Batchvarov V., Barc J., Crotti L., Bos J.M., Tester D.J., Muir A., Veltmann C. (2020). SCN5A mutation type and a genetic risk score associate variably with Brugada syndrome phenotype in SCN5A families. Circ. Genom. Precis. Med..

[23] Elliott P., Andersson B., Arbustini E., Bilinska Z., Cecchi F., Charron P., Dubourg  O., Kühl U., Maisch B., McKenna W.J. (2008). Classification of the cardiomyopathies: A position statement from the European Society Of Cardiology Working Group on Myocardial and Pericardial Diseases. Eur. Heart J..

[24] Bezzina C.R., Lahrouchi N., Priori S.G. (2015). Genetics of Sudden Cardiac Death. Circ. Res..

[25] Maron B.J., Maron M.S. (2013). Hypertrophic cardiomyopathy. Lancet.

[26] Li Q., Gruner C., Chan R.H., Care M., Siminovitch K., Williams L., Woo A., Rakowski H. (2014). Genotype-positive status in patients with hypertrophic cardiomyopathy is associated with higher rates of heart failure events. Circ. Cardiovasc. Genet..

[27] Ingles J., Goldstein J., Thaxton C., Caleshu C., Corty E.W., Crowley S.B., Dougherty K., Harrison S.M., McGlaughon J., Milko L.V. (2019). Evaluating the clinical validity of hypertrophic cardiomyopathy genes. Circ. Genom. Precis. Med..

[28] Ahmad F., McNally E.M., Ackerman M.J., Baty L.C., Day S.M., Kullo I.J., Madueme P.C., Maron M.S., Martinez M.W., Salberg L. (2019). Establishment of specialized clinical cardiovascular genetics programs: Recognizing the need and meeting standards: A scientific statement from the American Heart Association. Circ. Genom. Precis. Med..

[29] Bagnall R.D., Ingles J., Dinger M.E., Cowley M.J., Ross S.B., Minoche A.E., Lal S., Turner C., Colley A., Rajagopalan S. (2018). Whole genome sequencing improves outcomes of genetic testing in patients with hypertrophic cardiomyopathy. J. Am. Coll. Cardiol..

[30] Thomson K.L., Ormondroyd E., Harper A.R., Dent T., McGuire K., Baksi J., Blair E., Brennan P., Buchan R., Bueser T. (2019). Analysis of 51 proposed hypertrophic cardiomyopathy genes from genome sequencing data in sarcomere negative cases has negligible diagnostic yield. Genet. Med..

[31] Ho C.Y., Day S.M., Ashley E.A., Michels M., Pereira A.C., Jacoby D., Cirino A.L., Fox J.C., Lakdawala N.K., Ware J.S. (2018). Genotype and lifetime burden of disease in hypertrophic cardiomyopathy: Insights from the Sarcomeric Human Cardiomyopathy Registry (SHaRe). Circulation.

[32] Dewars E.R., Landstrom A.P. (2025). The Genetic Basis of Sudden Cardiac Death: From Diagnosis to Emerging Genetic Therapies. Annu. Rev. Med..

[33] Gollob M.H., Seger J.J., Gollob T.N., Tapscott T., Gonzales O., Bachinski L., Roberts R. (2001). Novel PRKAG2 mutation responsible for the genetic syndrome of ventricular preexcitation and conduction system disease with childhood onset and absence of cardiac hypertrophy. Circulation.

[34] Maron B.J., Roberts W.C., Arad M., Haas T.S., Spirito P., Wright G.B., Almquist A.K., Baffa J.M., Saul J.P., Ho C.Y. (2009). Clinical outcome and phenotypic expression in LAMP2 cardiomyopathy. JAMA.

[35] Elliott P., Baker R., Pasquale F., Quarta G., Ebrahim H., Mehta A.B., Hughes D.A., Anastasakis A., Autore C., Musumeci M.B. (2011). Prevalence of Anderson-Fabry disease in patients with hypertrophic cardiomyopathy: The European Anderson-Fabry Disease survey. Heart.

[36] Benson M.D., Waddington-Cruz M., Berk J.L., Polydefkis M., Dyck P.J., Wang A.K., Planté-Bordeneuve V., Barroso F.A., Merlini G., Obici L. (2018). Inotersen treatment for patients with hereditary transthyretin amyloidosis. N. Engl. J. Med..

[37] van Capelle C.I., Poelman E., Frohn-Mulder I.M., Koopman L.P., van den Hout J.M.P., Regal L., Cools B., Helbing W.A., van der Ploeg A.T. (2018). Cardiac outcome in classic infantile Pompe disease after 13years of treatment with recombinant human acid alpha-glucosidase. Int. J. Cardiol..

[38] Miron A., Lafreniere-Roula M.S., Fan C.P., Armstrong K.R., Dragulescu A., Papaz T., Manlhiot C., Kaufman B., Butts R.J., Gardin L. (2020). A validated model for sudden cardiac death risk prediction in pediatric hypertrophic cardiomyopathy. Circulation.

[39] Ingles J., Doolan A., Chiu C., Seidman J., Seidman C., Semsarian C. (2005). Compound and double mutations in patients with hypertrophic cardiomyopathy: Implications for genetic testing and counselling. J. Med. Genet..

[40] Towbin J.A. (2014). Inherited cardiomyopathies. Circ. J..

[41] Hershberger R.E., Hedges D.J., Morales A. (2013). Dilated cardiomyopathy: The complexity of a diverse genetic architecture. Nat. Rev. Cardiol..

[42] Petretta M., Pirozzi F., Sasso L., Paglia A., Bonaduce D. (2011). Review and metaanalysis of the frequency of familial dilated cardiomyopathy. Am. J. Cardiol..

[43] Kayvanpour E., Sedaghat-Hamedani F., Amr A., Lai A., Haas J., Holzer D.B., Frese K.S., Keller A., Jensen K., Katus H.A. (2017). Genotype-phenotype associations in dilated cardiomyopathy: Meta-analysis on more than 8000 individuals. Clin. Res. Cardiol..

[44] Van den Hoogenhof M.M.G., Beqqali A., Amin A.S., van der Made I., Aufiero S., Khan M.A.F., Schumacher C.A., Jansweijer J.A., van Spaendonck-Zwarts K.Y., Remme C.A. (2018). RBM20 mutations induce an arrhythmogenic dilated cardiomyopathy related to disturbed calcium handling. Circulation.

[45] Behere S.P., Weindling S.N. (2015). Inherited arrhythmias: The cardiac channelopathies. Ann. Pediatr. Cardiol..

[46] Ader F., De Groote P., Réant P., Rooryck-Thambo C., Dupin-Deguine D., Rambaud C., Khraiche D., Perret C., Pruny J.F., Mathieu-Dramard M. (2019). FLNC pathogenic variants in patients with cardiomyopathies: Prevalence and genotype-phenotype correlations. Clin. Genet..

[47] Gigli M., Merlo M., Graw S.L., Barbati G., Rowland T.J., Slavov D.B., Stolfo D., Haywood M.E., Dal Ferro M., Altinier A. (2019). Genetic risk of arrhythmic phenotypes in patients with dilated cardiomyopathy. J. Am. Coll. Cardiol..

[48] Haas J., Frese K.S., Peil B., Kloos W., Keller A., Nietsch R., Feng Z., Müller S., Kayvanpour E., Vogel B. (2015). Atlas of the clinical genetics of human dilated cardiomyopathy. Eur. Heart J..

[49] Jordan E., Hershberger R.E. (2021). Considering complexity in the genetic evaluation of dilated cardiomyopathy. Heart.

[50] Garnier S., Harakalova M., Weiss S., Mokry M., Regitz-Zagrosek V., Hengstenberg C., Cappola T.P., Isnard R., Arbustini E., Cook S.A. (2021). Genome-wide association analysis in dilated cardiomyopathy reveals two new players in systolic heart failure on chromosomes 3p25.1 and 22q11.23. Eur. Heart. J..

[51] Wahbi K., Yaou B., Gandjbakhch E., Anselme F., Gossios T., Lakdawala N.K., Stalens C., Sacher F., Babuty D., Trochu J.N. (2019). Development and Validation of a New Risk Prediction Score for Life-Threatening Ventricular Tachyarrhythmias in Laminopathies. Circulation.

[52] Barriales-VillaabJuan P.R., Ochoa J.P., Larrañaga-Moreiraa J.M., Salazar-Mendiguchíac J., Díez-López C., Restrepo-Córdoba M.A., Álvarez-Rubio J., Robles-Mezcua A., Olmo-Conesa M.C., Nicolás-Rocamora E. (2021). Risk predictors in a Spanish cohort with cardiac laminopathies. The REDLAMINA registry. Rev. Esp. Cardiol..

[53] Wahbi K., Behin A., Charron P., Dunand M., Richard P., Meune C., Vicart P., Laforêt P., Stojkovic T., Bécane H.M. (2012). High cardiovascular morbidity and mortality in myofibrillar myopathies due to DES gene mutations: A 10-year longitudinal study. Neuromuscul. Disord..

[54] Corrado D., van Tintelen P.J., McKenna W.J., Hauer R.N.W., Anastastakis A., Asimaki A., Basso C., Bauce B., Brunckhorst C., Bucciarelli-Ducci C. (2020). Arrhythmogenic right ventricular cardiomyopathy: Evaluation of the current diagnostic criteria and differential diagnosis. Eur. Heart J..

[55] Ackerman M.J., Priori S.G., Willems S., Berul C., Brugada R., Calkins H., Camm A.J., Ellinor P.T., Gollob M., Hamilton R. (2011). HRS/EHRA expert consensus statement on the state of genetic testing for the channelopathies and cardiomyopathies this document was developed as a partnership between the Heart Rhythm Society (HRS) and the European Heart Rhythm Association (EHRA). Heart Rhythm.

[56] Marcus F.I., Edson S., Towbin J.A. (2013). Genetics of arrhythmogenic right ventricular cardiomyopathy: A practical guide for physicians. J. Am. Coll. Cardiol..

[57] Kapplinger J.D., Landstrom A.P., Salisbury B.A., Callis T.E., Pollevick G.D., Tester D.J., Cox M.G.P.J., Bhuiyan Z., Bikker H., Wiesfeld A.C.P. (2011). Distinguishing arrhythmogenic right ventricular cardiomyopathy/dysplasia-associated mutations from background genetic noise. J. Am. Coll. Cardiol..

[58] Rigato I., Bauce B., Rampazzo A., Zorzi A., Pilichou K., Mazzotti E., Migliore F., Perazzolo Marra M., Lorenzon A., De Bortoli M. (2013). Compound and digenic heterozygosity predicts lifetime arrhythmic outcome and sudden cardiac death in desmosomal gene-related arrhythmogenic right ventricular cardiomyopathy. Circ. Cardiovasc. Genet..

[59] Zhou X., Chen M., Song H., Wang B., Chen H., Wang J., Wang W., Feng S., Zhang F., Ju W. (2015). Comprehensive analysis of desmosomal gene mutations in Han Chinese patients with arrhythmogenic right ventricular cardiomyopathy. Eur. J. Med. Genet..

[60] Quarta G., Muir A., Pantazis A., Syrris P., Gehmlich K., Garcia-Pavia P., Ward D., Sen-Chowdhry S., Elliott P.M., McKenna W.J. (2011). Familial evaluation in arrhythmogenic right ventricular cardiomyopathy: Impact of genetics and revised task force criteria. Circulation.

[61] Protonotarios A., Bariani R., Cappelletto C., Pavlou M., García-García A., Cipriani A., Protonotarios I., Rivas A., Wittenberg R., Graziosi M. (2022). Importance of genotype for risk stratification in arrhythmogenic right ventricular cardiomyopathy using the 2019 ARVC risk calculator. Eur. Heart J..

[62] Carrick R.T., Gasperetti A., Protonotarios A., Murray B., Laredo M., van der Schaaf I., Dooijes D., Syrris P., Cannie D., Tichnell C. (2024). A novel tool for arrhythmic risk stratification in desmoplakin gene variant carriers. Eur. Heart J..

[63] Chivulescu M., Lie Ø.H., Popescu B.A., Skulstad H., Edvardsen T., Jurcut R.O., Haugaa K.H. (2020). High penetrance and similar disease progression in probands and in family members with arrhythmogenic cardiomyopathy. Eur. Heart J..

[64] Bhonsale A., James C.A., Tichnell C., Murray B., Gagarin D., Philips B., Dalal D., Tedford R., Russell S.D., Abraham T. (2011). Incidence and predictors of implantable cardioverter-defibrillator therapy in patients with arrhythmogenic right ventricular dysplasia/cardiomyopathy undergoing implantable cardioverter-defibrillator implantation for primary prevention. J. Am. Coll. Cardiol..

[65] Fernandez-Falgueras A., Sarquella-Brugada G., Brugada J., Brugada R., Campuzano O. (2017). Cardiac Channelopathies and Sudden Death: Recent Clinical and Genetic Advances. Biology.

[66] Priori S.G., Wilde A.A., Horie M., Cho Y., Behr E.R., Berul C., Blom N., Brugada J., Chiang C.E., Huikuri H. (2013). HRS/EHRA/APHRS expert consensus statement on the diagnosis and management of patients with inherited primary arrhythmia syndromes: Document endorsed by HRS, EHRA, and APHRS in May 2013 and by ACCF, AHA, PACES, and AEPC in June 2013. Heart Rhythm.

[67] Grant A.O. (2001). Molecular biology of sodium channels and their role in cardiac arrhythmias. Am. J. Med..

[68] Wilde A.A.M., Amin A.S. (2018). Clinical spectrum of SCN5A mutations: Long QT syndrome, Brugada syndrome, and cardiomyopathy. J. Am. Coll. Cardiol. EP.

[69] Probst V., Wilde A.A., Barc J., Sacher F., Babuty D., Mabo P., Mansourati J., Le Scouarnec S., Kyndt F., Le Caignec C. (2009). SCN5A mutations and the role of genetic background in the pathophysiology of Brugada syndrome. Circ. Cardiovasc. Genet..

[70] Tadros R., Tan H.L., El Mathari S., Kors J.A., Postema P.G., Lahrouchi N., Beekman L., Radivojkov-Blagojevic M., Amin A.S., ESCAPE-NET Investigators (2019). Predicting cardiac electrical response to sodium-channel blockade and Brugada syndrome using polygenic risk scores. Eur. Heart J..

[71] Peltenburg P.J., Blom N.A., Vink A.S., Kammeraad J.A.E., Breur H., Rammeloo L.A.J., Wilde A.A.M., Clur S.A.B. (2020). In children and adolescents from Brugada syndrome-families, only SCN5A mutation carriers develop a type-1 ECG pattern induced by fever. Circulation.

[72] Moss A.J. (2003). Long QT syndrome. JAMA.

[73] Schwartz P.J., Crotti L., Insolia R. (2012). Long-QT syndrome: From genetics to management. Circ. Arrhythm. Electrophysiol..

[74] Krahn A.D., Laksman Z., Sy R.W., Postema P.G., Ackerman M.J., Wilde A.A.M., Han H.C. (2022). Congenital Long QT Syndrome. JACC Clin. Electrophysiol..

[75] Tawil R., Ptacek L.J., Pavlakis S.G., DeVivo D.C., Penn A.S., Ozdemir C., Griggs R.C. (1994). Andersen’s syndrome: Potassium-sensitive periodic paralysis, ventricular ectopy, and dysmorphic features. Ann. Neurol..

[76] Splawski I., Timothy K.W., Decher N., Kumar P., Sachse F.B., Beggs A.H., Sanguinetti M.C., Keating M.T. (2005). Severe arrhythmia disorder caused by cardiac L-type calcium channel mutations. Proc. Natl. Acad. Sci. USA.

[77] Jervell A., Lange-Nielsen F. (1957). Congenital deaf-mutism, functional heart disease with prolongation of the Q-T interval and sudden death. Am. Heart J..

[78] Crotti L., Odening K.E., Sanguinetti M.C. (2020). Heritable arrhythmias associated with abnormal function of cardiac potassium channels. Cardiovasc. Res..

[79] Wang D.W., Crotti L., Shimizu W., Pedrazzini M., Cantu F.D., Filippo P., Kishiki K., Miyazaki A., Ikeda T., Schwartz P.J. (2008). Malignant perinatal variant of long-QT syndrome caused by a profoundly dysfunctional cardiac sodium channel. Circ. Arrhythm. Electrophysiol..

[80] Adler A., Novelli V., Amin A.S., Abiusi E., Care M., Nannenberg E.A., Feilotter H., Amenta S., Mazza D., Bikker H. (2020). An international, multicentered, evidence-based reappraisal of genes reported to cause congenital long QT syndrome. Circulation.

[81] Crotti L., Spazzolini C., Tester D.J., Ghidoni A., Baruteau A.E., Beckmann B.M., Behr E.R., Bennett J.S., Bezzina C.R., Bhuiyan Z.A. (2019). Calmodulin mutations and life-threatening cardiac arrhythmias: Insights from the International Calmodulinopathy Registry. Eur. Heart J..

[82] Wang Q., Curran M.E., Splawski I., Burn T.C., Millholland J.M., VanRaay T.J., Shen J., Timothy K.W., Vincent G.M., de Jager T. (1996). Positional cloning of a novel potassium channel gene: KVLQT1 mutations cause cardiac arrhythmias. Nat. Genet..

[83] Curran M.E., Splawski I., Timothy K.W., Vincen G.M., Green E.D., Keating M.T. (1995). A molecular basis for cardiac arrhythmia: HERG mutations cause long QT syndrome. Cell.

[84] Wang Q., Shen J., Splawski I., Atkinson D., Li Z., Robinson J.L., Moss A.J., Towbin J.A., Keating M.T. (1995). SCN5A mutations associated with an inherited cardiac arrhythmia, long QT syndrome. Cell.

[85] Mazzanti A., Maragna R., Vacanti G., Monteforte N., Bloise R., Marino M., Braghieri L., Gambelli P., Memmi M., Pagan E. (2018). Interplay between genetic substrate, QTc duration, and arrhythmia risk in patients with long QT syndrome. J. Am. Coll. Cardiol..

[86] Mazzanti A., Trancuccio A., Kukavica D., Pagan E., Wang M., Mohsin M., Peterson D., Bagnardi V., Zareba W., Priori S.G. (2022). Independent validation and clinical implications of the risk prediction model for long QT syndrome (1-2-3-LQTS-Risk). EP Europace.

[87] Goldenberg I., Horr S., Moss A.J., Lopes C.M., Barsheshet A., McNitt S., Zareba W., Andrews M.L., Robinson J.L., Locati E.H. (2011). Risk for life-threatening cardiac events in patients with genotype-confirmed long-QT syndrome and normal-range corrected QT intervals. J. Am. Coll. Cardiol..

[88] Barsheshet A., Goldenberg I., O-Uchi J., Moss A.J., Jons C., Shimizu W., Wilde A.A., McNitt S., Peterson D.R., Zareba W. (2012). Mutations in cytoplasmic loops of the KCNQ1 channel and the risk of lifethreatening events: Implications for mutation-specific response to betablocker therapy in type 1 long-QT syndrome. Circulation.

[89] Dusi V., Pugliese L., De Ferrari G.M., Odero A., Crotti L., Dagradi F., Castelletti S., Vicentini A., Rordorf R., Li C. (2021). Left cardiac sympathetic denervation for long QT syndrome: 50 years’ experience provides guidance for management. JACC Clin. Electrophysiol..

[90] Schwartz P.J., Priori S.G., Spazzolini C., Moss A.J., Vincent G.M., Napolitano C., Denjoy I., Guicheney P., Breithardt G., Keating M.T. (2001). Genotype-phenotype correlation in the long-QT syndrome: Gene-specific triggers for life-threatening arrhythmias. Circulation.

[91] Schwartz P.J., Priori S.G., Locati E.H., Napolitano C., Cantu F., Towbin J.A., Keating M.T., Hammoude H., Brown A.M., Chen L.S. (1995). Long QT syndrome patients with mutations of the SCN5A and HERG genes have differential responses to Na^+^ channel blockade and to increases in heart rate. Implications for gene-specific therapy. Circulation.

[92] Bos J.M., Crotti L., Rohatgi R.K., Castelletti S., Dagradi F., Schwartz P.J., Ackerman M.J. (2019). Mexiletine shortens the QT interval in patients with potassium channelmediated type 2 long QT syndrome. Circ. Arrhythm. Electrophysiol..

[93] Nezu J., Tamai I., Oku A., Ohashi R., Yabuuchi H., Hashimoto N., Nikaido H., Sai Y., Koizumi A., Shoji Y. (1999). Primary systemic carnitine deficiency is caused by mutations in a gene encoding sodium ion-dependent carnitine transporter. Nat. Genet..

[94] Roussel J., Labarthe F., Thireau J., Ferro F., Farah C., Roy J., Horiuchi M., Tardieu M., Lefort B., François Benoist J. (2016). Carnitine deficiency induces a short QT syndrome. Heart Rhythm.

[95] Leenhardt A., Lucet V., Denjoy I., Grau F., Ngoc D.D., Coumel P. (1995). Catecholaminergic polymorphic ventricular tachycardia in children. A 7-year follow-up of 21 patients. Circulation.

[96] Medeiros-Domingo A., Bhuiyan Z.A., Tester D.J., Hofman N., Bikker H., van Tintelen J.P., Mannens M.M.A.M., Wilde A.A.M., Ackerman M.J. (2009). The RYR2encoded ryanodine receptor/calcium release channel in patients diagnosed previously with either catecholaminergic polymorphic ventricular tachycardia or genotype negative, exercise-induced long QT syndrome: A comprehensive open reading frame mutational analysis. J. Am. Coll. Cardiol..

[97] Priori S.G., Napolitano C., Tiso N., Memmi M., Vignati G., Bloise R., Sorrentino V., Danieli G.A. (2001). Mutations in the cardiac ryanodine receptor gene (hRyR2) underlie catecholaminergic polymorphic ventricular tachycardia. Circulation.

[98] Laitinen P.J., Brown K.M., Piippo K., Swan H., Devaney J.M., Brahmbhatt B., Donarum E.A., Marino M., Tiso N., Viitasalo M. (2001). Mutations of the cardiac ryanodine receptor (RyR2) gene in familial polymorphic ventricular tachycardia. Circulation.

[99] Arking D.E., Chugh S.S., Chakravarti A., Spooner P.M. (2004). Genomics in Sudden Cardiac Death. Circ. Res..

[100] Tester D.J., Arya P., Will M., Haglund C.M., Farley A.L., Makielski J.C., Ackerman M.J. (2006). Genotypic heterogeneity and phenotypic mimicry among unrelated patients referred for catecholaminergic polymorphic ventricular tachycardia genetic testing. Heart Rhythm.

[101] Swan H., Amarouch M.Y., Leinonen J., Marjamaa A., Kucera J.P., Laitinen-Forsblom P., Lahtinen A.M., Palotie A., Kontula K., Toivonen L. (2014). Gain-of-function mutation of the SCN5A gene causes exercise-induced polymorphic ventricular arrhythmias. Circ. Cardiovasc. Genet..

[102] Tester D.J., Ackerman J.P., Giudicessi J.R., Ackerman N.C., Cerrone M., Delmar M., Delmar M., McNally E.M., Wilde A.A.M., Priori S.G. (2019). Plakophilin-2 truncation variants in patients clinically diagnosed with catecholaminergic polymorphic ventricular tachycardia and decedents with exercise-associated autopsy negative sudden unexplained death in the young. JACC Clin. Electrophysiol..

[103] Roston T.M., Jones K., Hawkins N.M., Bos J.M., Schwartz P.J., Perry F., Sy R.W., James C.A., Bhonsale A., Tichnell C. (2018). Implantable cardioverter-defibrillator use in catecholaminergic polymorphic ventricular tachycardia: A systematic review. Heart Rhythm.

